# Measuring Implicit Sexual Response Biases to Nude Male and Female Pictures in Androphilic and Gynephilic Men

**DOI:** 10.1007/s10508-016-0725-3

**Published:** 2016-03-14

**Authors:** Liadh Timmins, Dermot Barnes-Holmes, Claire Cullen

**Affiliations:** 1 Department of Psychology, Institute of Psychiatry, King’s College London, 5th Floor, Bermondsey Wing, Guy’s Hospital Campus, London, SE1 9RT UK; 2 Department of Experimental, Clinical and Health Psychology, Ghent University, Ghent, Belgium; 3 School of Nursing & Human Sciences, Dublin City University, Dublin 9, Ireland

**Keywords:** Implicit measurement, Sexual orientation, Erotic preference

## Abstract

Snowden, Wichter, and Gray (2008) demonstrated that an Implicit Association Test and a Priming Task both predicted the sexual orientation of gynephilic and androphilic men in terms of their attraction biases towards pictures of nude males and females. For both measures, relative bias scores were obtained, with no information on the separate response biases to each target gender. The present study sought to extend this research by assessing both relative and individual implicit biases using the Implicit Relational Assessment Procedure (IRAP). An explicit measure screened for men with androphilic (*n* = 16) or gynephilic (*n* = 16) orientations on the dimensions of “sexual attraction,” “sexual behavior,” “sexual fantasies,” “hetero/gay lifestyle,” and “self identification.” The IRAP involved responding “True” or “False” to pictures of nude males and females as either attractive or unattractive. Participants were required to respond in a manner consistent with their reported sexual orientation for half of the IRAP’s test blocks and inconsistent for the other half. Response latencies were recorded and analyzed. The IRAP revealed a non-orthogonal pattern of biases across the two groups and had an excellent ability to predict sexual orientation with areas under the curves of 1.0 for the relative bias score and .94 and .95 for the bias scores for the male and female pictures, respectively. Correlations between the IRAP and explicit measures of sexual orientation were consistently high. The findings support the IRAP as a potentially valuable tool in the study of sexual preferences.

## Introduction

Researchers studying sexual orientation and sexual preference^1^ have begun to explore methods designed to measure so-called implicit attitudes. Such attitudes typically involve immediate, automatic (possibly unconscious), and non-declarative evaluations (De Houwer, 2006; De Houwer, Teige-Mocigemba, Spruyt, & Moors, 2009; Fazio & Olson, 2003; Gawronski, 2009), which are contrasted with the deliberate and controlled evaluative judgments (i.e., explicit attitudes) captured by self-report measures (Greenwald & Banaji, 1995; Nosek, 2007). There is an on-going debate concerning the nature of these two types of attitudes and how they operate and influence behavior (Gawronski & Bodenhausen, 2006; Rydell & McConnell, 2006; Wilson, Lindsey, & Schooler, 2000; for a recent review, see Hughes, Barnes-Holmes, & De Houwer, 2011). However, there exists a general consensus that measures of implicit and explicit attitudes are sensitive to “related but distinct constructs” (Nosek, 2005; but see Arkes & Tetlock, 2004). Critically, a growing body of evidence indicates that the two types of measure predict different types of behavior. Specifically, traditional self-report methodologies appear to predict intentional and controlled behaviors (Dovidio, Kawakami, Johnson, Johnson, & Howard, 1997; Dovidio, Kawakami, Smoak, & Gaertner, 2009), whereas scores obtained on implicit measures typically track spontaneous, immediate and, perhaps, more automatic responses and judgments (Friese, Hofmann, & Wanke, 2008; Galdi, Arcuri, & Gawronski, 2008; McConnell & Leibold, 2001).

This distinction may be of key importance for research into sexual preferences. While sexual orientation is often conceptualized as unidimensional in nature, there are likely multiple underlying constructs that determine human sexual behavior. Indeed, it is probable that explicit and implicit measures can tap into different classes of associated processes. For example, implicit measures may reflect fleeting thoughts and fantasies, visual interest in bodies of a particular sex, and/or arousal to those bodies, whereas explicit measures of sexual orientation may reflect desires to act on one’s arousal, strong sustained attractions to specific individuals, and/or other complex social information. Thus, implicit measures of sexual preference may tap into a unique aspect of sexual orientation that self-report methodologies cannot, which could present distinct patterns of responses within certain groups. For groups that display these divergent response patterns, either type of measure could prove to be a more accurate predictor of certain types of sexual behavior, sexual behavior within certain contexts, and/or sexual behavior altogether.

The first published study that sought to determine if sexual preference could be indexed with implicit measures (Snowden, Wichter, & Gray, 2008) employed two of the most well established methodologies, the Implicit Association Test (IAT) and a Priming Task (PT). Male participants who reported that they were either primarily androphilic or gynephilic completed both measures.

The critical parts of the IAT involved two types of computer-based tasks. In one task, participants were required to press the same button as quickly as possible if a picture of a nude male or a word indicating sexually attractive was presented (e.g., “arousing,” “erotic,” etc.); pressing a different button (as quickly as possible) was required if the computer presented a picture of a nude female or a word indicating sexually unattractive (e.g., “repulsive,” “repelling,” etc.). In the other task, the categorization responses were reversed; pressing one button for male pictures and *unattractive* and pressing the other button for female pictures and *attractive.* As predicted, the androphilic participants responded significantly more quickly when they were asked to categorize the male pictures with sexually attractive words and the female pictures with sexually unattractive words then vice versa (male with *unattractive* and female with *attractive*). Also as predicted, the gynephilic participants produced the opposite pattern to the androphilic participants; male pictures were categorized more rapidly with *unattractive* and females with *attractive* then vice versa. The relative difference in response latency between the two types of task was thus consistent with the participants’ self-reported sexual preferences. Furthermore, the IAT data successfully predicted self-reported sexual orientation with an area under the curve (AUC) of 0.97 and correlated strongly with a range of explicit measures of sexual preference (ranging from *r* = .72 to .80).

The other measure of implicit preference, the PT, also predicted self-reported sexual orientation, but with a slight drop in accuracy relative to the IAT (i.e., AUC = 0.86) and, once more, a range of correlations were obtained between the implicit and explicit measures (ranging from *r* = .49 to .56), although again these were weaker compared to the IAT. Finally, the two implicit measures correlated with each other (*r* = .59). Based on these findings, Snowden et al. (2008) concluded that “male sexual orientation to men or women can be indexed by implicit measures” (p. 563).

A limitation to the research reported by Snowden et al. (2008) is that one of their measures, the IAT, has a widely recognized weakness. Specifically, it provides only one *relative* bias score, which creates a lack of precision in determining the nature of the attitudes under study (see De Houwer, 2003). If, for example, participants responded more quickly on male-attractive and female-unattractive trials than on the reversed counterparts (i.e., male-unattractive and female-attractive), a number of interpretations are possible. For instance, participants may (1) have found males attractive and females aversive or (2) found both males and females attractive, but the former more so, or (3) found both males and females aversive, but the latter more so, or (4) found males attractive and females neither aversive nor attractive or (5) found females aversive and males neither aversive nor attractive.

This is particularly relevant for two reasons. Firstly, this severely restricts the IAT’s utility with bisexual individuals, given that bisexual individuals who experience strong, but not equal, sexual attraction to both males and females could be erroneously miscategorized as gynephilic or androphilic. Secondly, the IAT’s potential for exploring sexual aversion to the non-preferred gender in gynephilic and androphilic individuals is also limited. One might expect that such sexual aversion can be assumed, however self-report data suggests that while gynephilic males and females display aversion to sex with those of their non-preferred gender, androphilic females do not, and results for androphilic males are mixed (Freund, Langevin, Chamberlayne, Deosoran, & Zajac, 1974a; Freund, Langevin, Cibiri, & Zajac, 1973; Freund, Langevin, & Zajac, 1974b; Israel & Strassberg, 2009; Rullo, Strassberg, & Israel, 2010). Additionally, phallometric testing suggest that aversion does not exist at the level of genital arousal in androphilic or gynephilic men (Freund et al., 1973, 1974a, 1974b), whereas viewing time research suggests that it does exist in gynephilic men, but not in androphilic or gynephilic women (Israel & Strassberg, 2009; Rullo et al., 2010).

To measure implicit attitudes to individual types of stimuli, an alternative *non*-*relative* measure is thus required. In fact, a number of researchers have attempted to develop such non-relative tests, including, for instance, the Extrinsic Affective Simon Test (De Houwer, 2003), the Go/No-Go Association Task (Nosek & Banaji, 2001), and the Implicit Relational Assessment Procedure (IRAP) (Barnes-Holmes et al., 2006). As an aside, the PT employed by Snowden et al. (2008) could have yielded separate bias scores for male and female pictures but these were not reported in the article, presumably because they could not be compared meaningfully with the single relative IAT scores.

The present study sought to replicate and extend the research conducted by Snowden et al. (2008) by assessing both relative and individual implicit biases for male and female pictures using the IRAP. Research has shown that the IRAP (1) compares well with the IAT as a measure of individual differences (Barnes-Holmes, Murtagh, Barnes-Holmes, & Stewart, 2010c; Barnes-Holmes, Waldron, Barnes-Holmes, & Stewart, 2009), (2) is not easily faked (McKenna, Barnes-Holmes, Barnes-Holmes, & Stewart, 2007), (3) may be used as a measure of implicit self-esteem (Timko, England, Herbert, & Forman, 2010; Vahey, Barnes-Holmes, Barnes-Holmes, & Stewart, 2009), and (4) produces effects that indicate levels of bias not recorded with explicit measures (Barnes-Holmes, Murphy, Barnes-Holmes, & Stewart, 2010b; Dawson, Barnes-Holmes, Gresswell, Hart, & Gore, 2009; Power, Barnes-Holmes, Barnes-Holmes, & Stewart, 2009; Roddy, Stewart, & Barnes-Holmes, 2010).

One feature of the IRAP that was particularly important for the current study is that it consists of multiple trial-types, which, in principle, permits the assessment of more than one response bias (see Barnes-Holmes et al., 2010b). In the present research, each IRAP trial presented either a picture of a nude male or female as a label stimulus with either a positive (e.g., “arousing”) or negative (e.g., “repulsive”) target word. The IRAP thus allowed us to determine separate responses biases for the male and female pictures for gynephilic and androphilic participants, as well as an overall relative IRAP effect, similar to that reported by Snowden et al. (2008) for the IAT and PT.

The first aim of the current study was to replicate the findings reported by Snowden et al. (2008) with the IRAP. That is, we predicted that the overall relative IRAP effects would differ significantly between men who reported being primarily gynephilic versus androphilic and that this measure would successfully discriminate between the groups at a level similar to that obtained with the IAT and PT. We also predicted that the overall IRAP effect would yield similarly high correlations with the explicit measures of sexual orientation to those reported by Snowden et al. The second aim of the present research was more exploratory. Specifically, we sought to examine the separate IRAP effects generated by the male and female pictures by addressing the following five questions. First, would the IRAP effects for the male and female stimuli differ significantly for both the gynephilic and androphilic groups? Second, would both groups show significant IRAP effects consistent with their self-reported sexual orientation (i.e., an attraction bias for males only for the androphilic group and an attraction bias for females only for the gynephilic group?). Third, would both groups show significant IRAP effects consistent with aversion to their self-reported non-preferred gender? Fourth, would the two IRAP bias scores produce similar or different levels of predictive validity in terms of identifying the sexual orientation of the participants? Fifth, would the IRAP bias scores correlate with the explicit measures employed in the study?

## Method

### Participants

Given that the current study was a “first test” of the validity and utility of the IRAP as a measure of sexual orientation, participants were 16 gynephilic men (*M* age = 23.8 years; range, 18–54) and 16 androphilic men (*M* age = 22.8; range, 18–39). Gynephilic and androphilic men tend to display category-specific sexual responses at both a subjective and genital arousal level, whereas this is less so the case in gynephilic women (Chivers, Rieger, Latty, & Bailey, 2004; Chivers, Seto, & Blanchard, 2007; Chivers, Seto, Lalumiére, Laan, & Grimbos, 2010). This makes gynephilic and androphilic men ideal to test the discriminability of the IRAP at this early stage.

Gynephilic participants were students of Maynooth University. Androphilic participants were recruited through the Lesbian, Gay, Bisexual and Transgender society at Maynooth University and via snowball sampling through those participants. Consistent with Snowden et al. (2008), gynephilic men were operationally defined as men with a relatively stable preference for sexual partners of the opposite gender and androphilic men were defined as men with a relatively stable preference for sexual partners of the same gender. Such preference was confirmed by a modified version of the Klein Sexual Orientation Grid (KSOG) (Klein, 1993; Klein, Sepekoff, & Wolf, 1985), which showed all participants to be either primarily gynephilic or androphilic (see next section for details). Volunteers received a chocolate brownie for their participation, but no other rewards or incentives were offered.

### Measures

An information and consent booklet was used to brief participants. This consisted of the following brief summary of the general nature of the study, as well as reproductions of the 10 nude stimuli to be used in the study and a copy of the consent form: “Our research investigates cognitive processes that are used in decisions that involve memory. We are seeking to develop and test theories of cognitive processes that occur inside and outside of awareness in the routine use of memory. In this case, the cognitive processes involved in making decisions about the sexual appeal of males and females are being investigated. As such, nude images of both males and females will be presented multiple times during the experiment. Your identity as a subject is confidential. Further, you are free to discontinue participation at any time, without penalty.”

The same five male and four of the five female picture stimuli used by Snowden et al. (2008), taken from the International Affective Picture System (IAPS) (Lang, Bradley, & Cuthbert, 1997), were employed in the current study (male picture numbers: 4460, 4500, 4534, 4550, 4561; female picture numbers: 4141, 4142, 4210, 4240). A fifth female picture (picture number: 4235) was chosen from the IAPS in lieu of the original fifth picture used by Snowden et al. (picture number: 4332) due to its unavailability. All pictures chosen by Snowden et al. were picked for their erotic, but not pornographic content, as was the fifth female picture in the current study; subjects in the pictures were completely or almost completely nude, while not visibly sexually aroused nor engaged in sexual activity.

The five word stimuli pertaining to “sexually attractive” originally used by Snowden et al. (2008) were also employed in the current study (i.e., “arousing,” “erotic,” “attractive,” “sensual,” and “exciting”). However, only four of the five original words pertaining to “sexually unattractive” were used (i.e., “repulsive,” “repelling,” “repugnant,” and “repellent”). During pilot testing, the fifth word (“forbidding”) was deemed to be ambiguous in the context of the IRAP because it had moralistic connotations, which applied to all of the nude images (both male and female) irrespective of sexual orientation. Consequently, the word “awful” was used in its place.

The explicit attitude measures consisted of the semantic differential measures used by Snowden et al. (2008), as well as a version of the KSOG, modified to reflect the results of a factor analysis of the instrument (Weinrich et al., 1993). The KSOG consisted of five dimensions of sexual orientation (sexual attraction, sexual behavior, sexual fantasies, hetero/gay lifestyle, and self identification), all of which were assessed on a seven point scale across two temporal dimensions (past, defined as up to a year ago, and present, defined as the last 12 months), as well as a third dimension of ideality (defined as what the participant would like). Higher scores indicated a more androphilic attitude and lower scores indicated a more gynephilic attitude. This resulted in a total of 15 scores of sexual orientation (Cronbach’s alpha for present study = .98).

Mean scores were rounded off to the nearest whole number, and this final score was used as a screening measure for the study (KSOG scores were not rounded off to the nearest whole number for anything other than this screening). Scores of 1–3 were deemed to represent an overall sexual preference for women, scores of 5–7 an overall sexual preference for men, and a score of 4 a relative lack of definite preference for either men or women. No participant had a score of 4 and all participants’ scores were in accordance with their reported sexual orientation, with gynephilic individuals scoring between 1 and 3 and androphilic individuals scoring between 5 and 7.

The semantic differentials involved two identical sets of six bipolar Likert scales, one for the concept “sex with men is (to me)” and another for the concept “sex with women is (to me).” The Likert scales each had a pair of opposite adjectives at either end. These pairs were “good/bad,” “beautiful/ugly,” “pleasant/unpleasant,” “exciting/boring,” “nice/awful,” and “attractive/unattractive.” The scales ranged from 1 to 7, with 4 as the neutral point. Higher numbers indicated a more favorable attitude, except in the case of the “pleasant/unpleasant” scales, in which the labels were reversed (to control for repetitive responding). The data for this scale were recoded before the data analysis to render the direction of effects consistent with the other data.

The IRAP software, which was run on a standard personal computer, was written by the second author and is available upon request. Participants completed the study alone in a small quiet room free of distraction.

### Procedure

Participants were informed that the study would consist of a short questionnaire about their sexual orientation and behavior, followed by a computerized task. For ethical reasons, participants were also informed that both were intended as measures of sexual preference, but that the data were being collected anonymously and as such could not be directly traced to them. In addition, the participants were informed they had the right to cease participation at any time, as well as retract their data afterwards. Participants who inquired further as to how the IRAP measures sexual preference were informed that it determines it based on their responses to the stimuli, but no more specific information was given.

If participants confirmed they were willing to continue, they were presented with the information and consent booklet, described previously. Participants were then offered a minimum of a 24-h “change-of-mind” period to allow them to reconsider their participation. To avoid inconveniencing participants unnecessarily, those who wished to continue with the study immediately were allowed to do so.

No participants chose to cancel their participation after the change-of-mind period, and, upon their return, they were again presented with the booklet and asked to sign the consent form if they wished to continue. Having signed, participants then completed the explicit measures (the KSOG and semantic differentials).

Subsequently, participants were seated in front of the computer, which presented the instructions and stimuli and recorded all responses. The IRAP software began by presenting a set of instructions, which explained the IRAP task using illustrative examples of the different types of trials, and giving a detailed account of what participants were required to do.

**Fig. 1:**
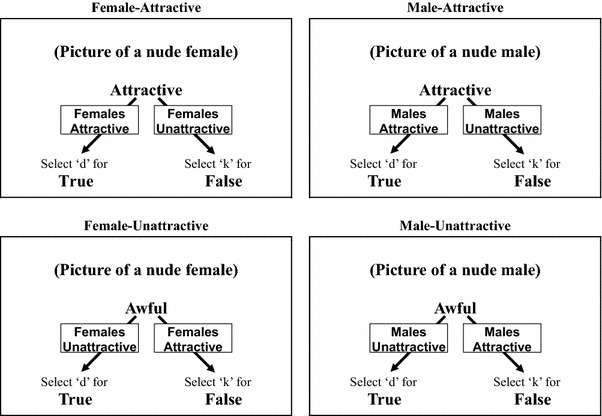
The four IRAP trial-types. The nude picture stimuli, word stimuli and response options (“True” and “False”) appeared simultaneously on each trial. *Arrows* with superimposed text show which responses indicate which bias (*text* and *arrows* did not appear on screen)

The IRAP was presented in blocks of 40 trials. Trials consisted of the simultaneous presentation of either a male or female nude picture stimulus at the top of the screen, either an attractive or unattractive word stimulus in the middle of the screen and response options of “True” and “False” in the bottom left- and right-hand corners, with the instructions “Press ‘D’ for” and “Press ‘K’ for” directly above the left and right response options, respectively. The left–right positioning of the two response options, and therefore the keys required to select them, varied randomly across trials, with the constraint that they could not appear in the same positions across more than three successive trials. The different combinations of male/female and positive/negative words resulted in four possible trial types: Male-Attractive, Male-Unattractive, Female-Attractive, and Female-Unattractive (see Fig. 1).

During each block, participants had to respond in accordance with one of two rules, regardless of their own personal feelings: (1) “all females are attractive and all males are unattractive” (defined as a *female*-*attractive* block) or (2) “all males are attractive and all females are unattractive” (defined as a *male*-*attractive* block). The trials were presented quasi-randomly with the constraint that each of the four trial-types appeared 10 times within each 40-trial block, all 10 picture and 10 word stimuli were presented twice within each block and the same trial-type was not presented across successive trials.

Choosing the response option deemed correct cleared the screen for a 400 ms inter-trial interval and then the next trial was presented. If the incorrect response option was chosen, a red X appeared directly underneath the target word and remained there until the participant chose the correct response option. If a participant failed to respond within 2000 ms from the start of a trial, the words “Too Slow” appeared towards the center bottom of the screen and remained there until the participant chose one of the response options.

Participants were first presented with a set of two practice blocks. Participants were required to achieve an accuracy criterion of ≥80 % correct responses and a median response latency of ≤2000 ms. If these criteria were achieved, participants were then exposed to fixed set of six test blocks. If they were not achieved, the practice blocks were repeated until they were. Participants were not required to achieve any performance criteria during the test blocks in order to proceed. However, accuracy and latency feedback were presented at the end of each block to encourage participants to maintain the performance criteria achieved during the practice blocks.

Blocks were presented in one of two possible sequences, each alternating between the presentation of a *female*-*attractive* and a *male*-*attractive* block. In one sequence, participants were first exposed to a *female*-*attractive* block, whereas in the other sequence participants were first exposed to a *male*-*attractive* block. Block sequence was counterbalanced across participants. Upon completion of the IRAP, participants were thanked and debriefed and reminded that if they wished they could still revoke their data.

### Data Analysis

The primary datum for the IRAP was response latency defined as time in milliseconds from the onset of a test trial until the emission of a correct response. Consistent with the majority of published IRAP studies, individual response latency data were transformed into *D*-IRAP scores (see Barnes-Holmes, Barnes-Holmes, Stewart, & Boles, 2010a) using an adaptation of the Greenwald, Nosek, and Banaji (2003) *D*-algorithm.

The *D*-algorithm produced a *D*-IRAP score for each of the four trial types. For the two female trial type scores, a positive score indicated an attraction bias and a negative score indicated an aversion bias, whereas for the two male trial type scores a negative score indicated an attraction bias and a positive score indicated an aversion bias. The mean of the two female trial type scores constituted the female pictures *D*-IRAP score, and the mean of the two male trial type scores multiplied by −1 constituted the male pictures *D*-IRAP score. A positive score thus indicated an attraction bias, whereas a negative score indicated the opposite. The mean of the four trial-types scores constituted the overall mean *D*-IRAP score. A positive score thus indicated a gynephilic bias (i.e., stronger attraction to female than male pictures) whereas a negative score indicated a androphilic bias (i.e., stronger attraction to male than female pictures).

## Results

### Implicit Measure

A preliminary analysis showed that block sequence (*female*-*attractive*-*first* versus *male*-*attractive*-*first*) did not have a significant effect on performance; hence, this variable was removed from subsequent analyses.

**Fig. 2:**
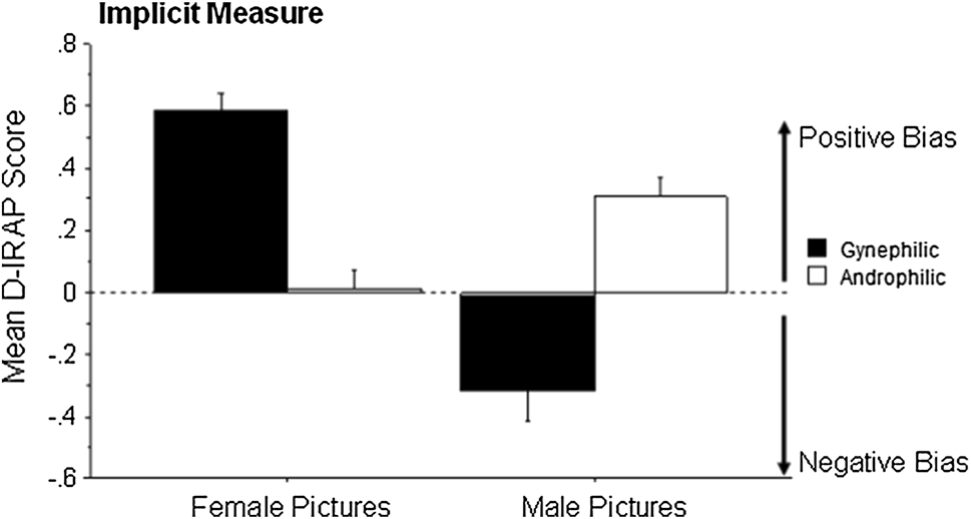
Mean *D*-IRAP scores with standard *error bars* for female picture and male picture trial-types for Gynephilic and Androphilic participants. A positive score indicates a positive bias (attraction) and a negative score indicates a negative bias (aversion)

The mean *D*-IRAP scores for the male and female pictures are shown in Fig. 2. The scores for the female pictures were .59 (*SE* = .06) for the gynephilic participants and .01 (*SE* = .06) for the androphilic participants; for the male pictures, the scores were −.31 (*SE* = .10) for the gynephilic participants and .31 (*SE* = .06) for the androphilic participants. The gynephilic participants thus showed a strong positive (attraction) bias towards the female pictures with a negative (aversion) bias towards the male pictures. In contrast, the androphilic group showed a strong positive bias towards the male pictures, but virtually no directional bias for the female pictures.

The mean *D*-IRAP scores for the male and female pictures were subjected to a 2 × 2 mixed repeated measures analysis of variance (ANOVA) with sexual orientation as a between-participant variable and IRAP trial-type (male versus female) as the within participant variable. The ANOVA yielded a significant interaction effect, *F*(1, 30) = 59.07, *p* < .0001, *η*_*p*_^2^ = .66. Four *t* tests were used to explore the interaction. Two unpaired *t* tests showed a significant effect for both the female trial-type, *t*(30) = 7.21, *p* < .0001, *d* = 2.55 and the male trial-type, *t*(30) = −5.29, *p* < .0001, *d* = 1.89. Two paired *t* tests showed a significant effect for both the gynephilic group, *t*(15) = 6.7, *p* < .0001, *d* = 1.70, and androphilic group, *t*(15) = −3.8, *p* = .0018, *d* = .95. Four one sample *t* tests were conducted to determine if the *D*-IRAP scores differed significantly from zero. Both scores for the gynephilic group were significant: female pictures, *t*(15) = 10.49, *p* < .0001, *d* = 5.42; male pictures, *t*(15) = −3.15, *p* = .0066, *d* = 1.63. The male picture scores for the androphilic group also differed significantly from zero, *t*(15) = 4.95, *p* = .0002, *d* = 2.56, but the female picture scores did not.

The overall mean *D*-IRAP score was −.15 (*SE* = .04) for the androphilic group and .45 (*SE* = .07) for the gynephilic group, and this difference proved to be significant, *t*(30) = 7.69, *p* < .0001, with a very large effect size (*d* = 2.72).

### Prediction of Sexual Orientation

A main aim of the current research was to determine if an implicit measure could be used to differentiate between the sexual preferences of gynephilic and androphilic men, and to measure this predictive ability. As such, the same signal detection test employed by Snowden et al. was used here, which involved constructing the Receiver Operator Characteristic (ROC). A ROC is a graph in which the probability of a true positive, or a “hit,” is plotted against the probability of a false positive or a “false alarm” (Fawcett, 2006). From this, the AUC can be calculated, which essentially is the statistical likelihood that a randomly chosen member of the “positive” group (in this case, gynephilic participants) will have a higher score than a randomly chosen member of the “negative” group (in this case androphilic individuals). Therefore, a test with perfect ability to predict group membership would have an AUC = 1.0, and a test with no ability to detect group membership would have an AUC = ~0.5.

**Fig. 3:**
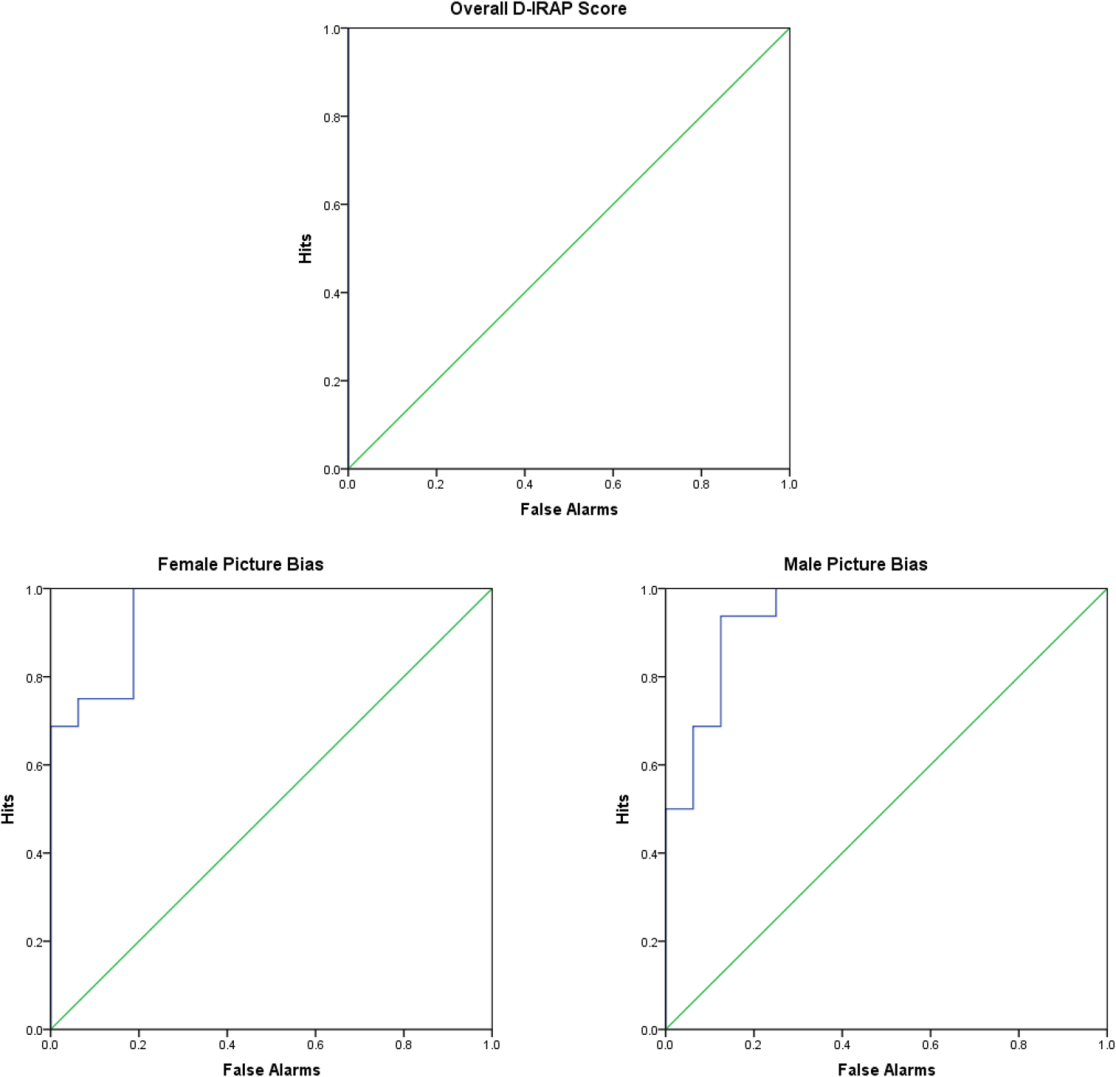
Receiver operating characteristics of the ability of the overall mean *D*-IRAP, female and male picture bias scores to predict sexual orientation. The *straight diagonal lines* represent chance level. The area under the curve (AUC) is 1.0 (*p* < .001) for the overall *D*-IRAP scores, 0.95 (*p* < .001) for the female picture bias scores and 0.94 (*p* < .001) for the male picture bias scores

The ROCs for the overall *D*-IRAP score, female picture, and male picture scores are shown in Fig. 3. Using the overall score, the IRAP proved to be a perfect predictor of sexual orientation, with an AUC = 1.0 (*p* < .001). The ROC analysis for the female and male picture scores showed similarly strong abilities to identify sexual orientation, although these were not perfect, with female AUC = 0.95 (*p* < .001) and male AUC = 0.94 (*p* < .001).

### Differential Preference Scales

To make the semantic differential measures compatible with the overall *D*-IRAP scores, the scores for the “Sex with Men” Likert scales were subtracted from the scores for the “Sex with Women” Likert scales. Positive scores thus indicate a preference for women and negative scores a preference for men. These measures are referred to as Differential Preference Scales (DPSs), and the overall means for these data along with the overall means from the two separate Likert scales are shown in Table 1.

**Table 1:** Descriptive and inferential statistics for the explicit measures

	Group				Between groups comparison	
	Gynephlic		Androphilic			
	*M*	*SD*	*M*	*SD*	*t*	*d*
Measure						
Good						
Preference	4.4	1.7	−3.7	1.4	14.41***	5.09
Sex w/women	6.8	0.4	3.1	1.2		
Sex w/men	2.4	1.6	6.8	0.5		
Beautiful						
Preference	3.6	1.1	−2.4	1.8	11.42***	4.04
Sex w/women	6.0	0.9	2.8	1.4		
Sex w/men	2.4	1.3	5.3	1.4		
Pleasant						
Preference	4.7	1.7	−3.6	1.8	13.64***	4.82
Sex w/women	6.9	0.3	2.8	1.4		
Sex w/men	2.2	1.6	6.4	1.1		
Exciting						
Preference	2.7	1.6	−3.6	1.7	10.85***	3.84
Sex w/women	6.3	1.0	2.9	1.6		
Sex w/men	3.6	1.0	6.5	0.6		
Nice						
Preference	3.9	1.3	−2.9	2.0	11.44***	4.05
Sex w/women	6.9	0.3	3.4	1.6		
Sex w/men	3.0	1.3	6.3	1.1		
Attractive						
Preference	4.9	1.2	−3.4	2.0	13.99***	4.95
Sex w/women	6.8	0.5	2.8	1.6		
Sex w/men	1.9	1.2	6.2	1.3		
KSOG	1.5	0.4	5.9	0.5	27.47***	10.03

Range for preference differentials, −6.0 to 6.0. Range for “Sex with Women” and “Sex With Men” differentials, 1.0–7.0. Range for KSOG-m, 1.0–7.0

*** *p* < .0001

In all cases, the direction of the effects was consistent with predicted group differences. The data for the DPSs were entered into a 2 × 6 multivariate analysis of variance (MANOVA) with sexual orientation as a between-participant variable and the 6 DPS scores as within participant variables. The MANOVA yielded a significant main effect, *F*(1, 30) = 57.26, *p* < .001, *ηp* ^*2*^ = .93. Six unpaired *t* tests were used to explore the nature of this main effect and all 6 *t* tests were significant (see Table 1).

Twelve one-sample *t* tests were used to determine if the results for the DPSs differed significantly from zero (i.e., a neutral preference). All 12 *t* tests (six for the gynephilic group and six for the androphilic group) proved to be highly significant (all *ps* < .0001). A further 24 one-sample *t* tests were employed to determine if the “Sex with Women” and “Sex with Men” Likert ratings differed significantly from 4 (i.e., a neutral preference). For the gynephilic group, 11 of the *t* tests were significant (*p*s < .01, *d*s > 1.5). Similarly, for the androphilic group, 11 of the *t* tests were significant (*p*s < .02, *d*s > 1.4).

### Klein Sexual Orientation Grid

The data for the KSOG were similarly entered into an unpaired *t* test. As would be expected, given that the KSOG was used as a screening measure, it too produced a large and significant difference (see Table 1).

### Relationship Between the Measures

Pearson correlation coefficients were computed to examine the relationships between the variables (see Table 2). The Overall *D*-IRAP scores were correlated with the DPSs and the two *D*-IRAP picture bias scores were correlated with their corresponding gender-specific Likert scales (i.e., male picture bias scores with “Sex with Men” ratings and female picture bias scores with “Sex with Women” ratings). The three *D*-IRAP measures were also correlated with the KSOG. All correlations with the Overall *D*-IRAP scores were very high and significant, ranging between *r* = .77 (“Pleasant” Differential Preference Scale) and *r* = .84 (“Good” Differential Preference Scale). All of the correlations between each of the two picture bias scores and the explicit measures were also significant, although in general they were slightly weaker than the overall *D*-IRAP correlations. Finally, a Pearson correlation coefficient was calculated between the two *D*-IRAP picture bias scores and this proved to be negative and significant, *r* = −.65, *p* < .0001 (the correlation was negative because an attraction bias to one gender predicted an aversion bias to the opposite gender).

**Table 2:** Correlations (Pearson *r*) between *D*-IRAP scores and explicit measures

	Overall *D*-IRAP score	Female picture bias	Male picture bias
Measure			
Good			
Preference	.84***		
Sex w/women		.79***	
Sex w/men			.70***
Beautiful			
Preference	.82***		
Sex w/women		.68***	
Sex w/men			.56**
Pleasant			
Preference	.77***		
Sex w/women		.71***	
Sex w/men			.55**
Exciting			
Preference	.82***		
Sex w/women		.69***	
Sex w/men			.76***
Nice			
Preference	.82***		
Sex w/women		.62***	
Sex w/men			.68***
Attractive			
Preference	.80***		
Sex w/women		.72***	
Sex w/men			.57**
KSOG	.82***	.81***	.69***

** *p* < .001; *** *p* < .0001

## Discussion

The results of the current study supported Snowden et al.’s (2008) conclusion that implicit measures can be used to distinguish between men of different sexual orientations. In addition, the data indicated that the IRAP had a level of predictive validity that compared favorably with the levels reported by Snowden et al. for the IAT and the PT. Furthermore, high correlations between the IRAP and the explicit measures were found, which again compared favorably with those reported for the IAT and the PT, which Snowden et al. pointed out were already higher than all previously published comparisons. Critically, the level of predictive validity and correlation with explicit measures remained high even when bias scores were calculated only using the implicit responses to either the male or female pictures. The current findings thus supported the conclusion that the sexual orientation of gynephilic and androphilic men may be distinguished based not only on *relative* preference scores for male and female erotic stimuli, but also on scores obtained separately for each gender.

Although implicit responses to both types of stimuli (male and female) yielded very high levels of predictive validity, the pattern of biases shown for the two types of stimuli for the androphilic and gynephilic men was not strictly orthogonal. Specifically, the gynephilic group showed clear attraction and aversion biases for the female and male pictures, respectively, whereas the androphilic group only showed the opposite pattern for the male stimuli. Interestingly, the female nudes produced a near neutral IRAP effect for the androphilic group. It is worth noting that the non-orthogonal pattern for the IRAP was somewhat reflected in the KSOG scores, in which the mean for the androphilic group was 5.9 (i.e., 1.1 away from maximum exclusivity) whereas the mean for the gynephilic group was 1.5 (i.e., 0.5 away from maximum exclusivity). On balance, five of the six one-sample *t* tests for the Likert scales were significantly different from neutral in a negative direction for the androphilic group when rating “Sex with Women.” Thus, although the androphilic group produced self-reports that suggested a lower level of exclusivity relative to the gynephilic group in terms of general sexual preference, the ratings of the androphilic group with respect to sexual attraction to women were far from neutral. How might we account for this apparent divergence between the implicit and explicit measures?

One important factor that might have served to reduce the implicit female picture bias for the androphilic group to near zero is the life-long repeated media presentations of women as sexual objects and as possessing great sexual appeal. For example, in advertising not only is the sexual appeal of women portrayed more often than men, but also the female models used for this purpose tend to be more attractive, more slender, and younger than males who are used for their sex appeal (Lin, 1998). Almost daily exposure to this focus on females as attractive, sexual beings may thus have impacted upon the automatic responses to the female stimuli. For the gynephilic group, the portrayal of women as primarily sexual would only serve to support those automatic responses that were consistent with self-reported sexual orientation. In the case of the androphilic group, however, constant exposure to females as sexual in the wider culture may influence automatic responses in a manner that diverges from self-reported levels of attraction to the opposite sex.

Also of note is that androphilic men are more likely to have had sexual experience with their non-preferred gender than gynephilic men (Layte et al., 2006). It is possible that this exposure may have affected the implicit bias in the androphilic men in this sample or even vice versa. However, without non-relativistic information on numbers of same and opposite sex partners, this hypothesis was untestable using the current data set.

Of course, both explanations remain speculative, but there is considerable evidence that implicit measures are sensitive to the impact of evaluative conditioning (Olson & Fazio, 2001, 2002) and other iterative learning procedures (Cullen, Barnes-Holmes, Barnes-Holmes, & Stewart, 2009; Hughes & Barnes-Holmes, 2011). Consequently, exposure to women in a sexual context in the above-described ways may indeed impact on measures of implicit sexual response biases. In any case, the fact that the IRAP yielded an effect that diverged somewhat from the explicit ratings serves to highlight the potential utility of employing such measures in the investigation of sexual orientation.

Of key interest would be to investigate to what degree implicit sexual orientation as measured by the IRAP predicts sexual behavior and arousal, especially in the context of a discrepancy with explicit attitudes, such as that displayed by the androphilic men in this study. As discussed previously, explicit and implicit measures may tap into distinct aspects of sexual orientation. Identifying the conditions under which the IRAP and explicit measures predict other aspects of sexuality and which aspects each predict could help tease out what dimensions of sexual orientation these two types of measures assess. For example, if implicit measures predicted genital arousal to novel images and opportunistic sexual interaction, but explicit measures predicted deliberately sought interactions and long term sexual interest in particular persons, one might conclude that the IRAP taps into immediate sexual arousal, whereas explicit measures tap into desires to act on one’s arousal or arousal that may be sustained or induced at later stages of sexual interaction. An additional area of interest would be sexual behavior in cases of neither attraction nor aversion. Theoretically, the former should motivate sexual behavior, and the latter should deter it, but if an individual lacks either response to a particular gender they could be motivated to engage in such behaviors with members of that gender by other factors, such as curiosity, sensation seeking and/or miscellaneous social benefits.

A limitation of the current study was that participation was restricted to gynephilic and androphilic men only. As such, the current findings tell us little about androphilic women, gynephilic women and bisexual individuals. The implicit sexual responses of women would be of particular interest, given that gynephilic women typically do not display a category specific response pattern on genital measures (Chivers et al., 2007, 2010). Since the IRAP is an objective measure, one might predict that it would show a similar response pattern to that exhibited by genital arousal measures. However, the IRAP is also essentially a verbal measure (Barnes-Holmes et al., 2010a), which suggests that the construct it measures is likely more directly related to explicit self-reports than genital measures are. As such, gynephilic women may yield category-specific IRAP scores concordant with their reported sexual orientation. Such a finding would imply that while gynephilic women are category-specific at a verbal or cognitive level, this is not the case in terms of genital arousal, which would in turn help explain why the majority of women consider themselves to be gynephilic despite what research utilizing genital arousal measures seems to suggest.

Likewise, the IRAP could be used to investigate the phenomenal nature of bisexuality. Notably, some bisexual men display a category-specific response pattern on genital arousal measures (Rieger, Chivers, & Bailey, 2005, but see Rosenthal, Sylva, Safron, & Bailey, 2011). If those who exhibit this pattern on genital measures display significant attraction biases to both males and females on the IRAP, this would suggest that bisexuality can exist in men at the verbal or cognitive level even when it cannot be detected using phallometry. Similarly, bisexual individuals that display dual attraction biases may constitute multiple subgroups, such that some may possess equally strong biases for each gender and others may have a bias that is more pronounced for one gender or the other.

A further limitation was that the participants were informed that the research involved measures of sexual orientation and were asked only to volunteer if they felt they had a strong preference for one gender over the other. As such, participants were relatively open about their sexuality and sexual matters generally and it seems unlikely that any participant would have been trying to hide or fake his sexual orientation. The current findings do not, therefore, indicate if the IRAP could be used to tap into sexual response biases when participants are attempting to engage in dissimulation or lack awareness of such biases. Indeed, a true test of its utility in that regard would require participants for whom their actual sexual attraction patterns are inconsistent with those they explicitly report and ideally the results would also be compared with phallometric testing. Nevertheless, previous research has indicated that the IRAP is difficult to fake (McKenna et al., 2007; see also Barnes-Holmes et al., 2010a), and psychologically sensitive biases not revealed by explicit measures have been obtained with the IRAP (Barnes-Holmes et al., 2010b; Dawson et al., 2009; Roddy et al., 2010). Consequently, further research with the IRAP to determine its resistance to faking (or sensitivity to unconscious biases) in the context of sexual preferences certainly seems worthwhile.

A recent attempt to develop the IRAP as a forensic measure for distinguishing the implicit sexual responses of child sex offenders from non-offenders showed moderate predictive validity (Dawson et al., 2009). It was suggested that the accuracy of the IRAP may have been compromised by the heterogeneity of the offending group (Gudjonsson & Sigurdsson, 2000), and a more precise understanding of these differences may lead to more effective means of their assessment (Robertiello & Terry, 2007). Indeed, the current study’s success suggests that when sexual preference is more clearly defined, distinguishing between the implicit sexual responses of groups with different sexual preferences may be achieved with very high levels of accuracy using the IRAP, as well as the IAT and PT. It is worth noting, however, that other variables may play a role here. For example, words rather than pictures were employed with the IRAP in the Dawson et al. study, and the latency criterion was set at 3000 rather than 2000 ms (the latter was used in the current study). Pictures, and particularly nudes, may elicit relatively strong sexual response biases and recent research indicates that stronger and more reliable IRAP effects are produced when a shorter response latency criterion is employed (Barnes-Holmes et al., 2010b). Numerous variables will thus require systematic analysis in the search for increasingly accurate measures of sexual preference using the IRAP and indeed other measures of implicit attitudes (see O’Ciardha & Gormley, 2009, 2012).

As mentioned previously, one advantage the IRAP has over the IAT is its ability to measure biases in a non-relative manner. It is worth noting that this disadvantage can be worked around by replacing one of the target stimulus sets with a set of presumably neutral stimuli, thereby theoretically creating a non-relative measure of implicit biases to a single concept. This has been done specifically with the IAT in order to measure individual implicit sexual responses to male and female stimuli (Snowden & Gray, 2013). This was performed as a follow up to an IAT which measured sexual responses relatively. However, this solution still has some inherent issues. Firstly, research suggests that exposure to a single IAT results in vulnerability to faking in subsequent IATs, even without explicit instructions on how (Fiedler & Bluemke, 2005), which may limit this form of the IAT’s ability to index an individual’s full profile of implicit sexual responses to male and female stimuli.

Secondly, unless another non-relative implicit measure is used to assess them in advance, the neutrality of the replacement stimuli is somewhat of an a priori assumption, which can complicate interpretation. For example, according to the mentioned IATs utilized by Snowden and Gray (2013), gynephilic males appeared to be more sexually attracted to female pictures versus male pictures, equally sexually attracted to male pictures versus neutral pictures, and more sexually attracted to female pictures versus neutral pictures. This was interpreted as category-specific attraction to the female stimuli, as it is quite unlikely that these participants would display sexual attraction to the neutral stimuli at a group level. However, it was unclear whether these men had no biases to both the male and neutral stimuli or aversion biases to both the male and neutral stimuli, both of which could be possible. Intuitively, a neutral score implies a neutral attitude to the male stimuli on the male versus neutral IAT; however, the data from the IRAP imply that gynephilic men may have an implicit aversion bias to male stimuli. Follow up research could test this by administering the IRAP and these IATs to gynephilic male participants, and indeed the IRAP could potentially be used to test the validity of the male versus neutral and female versus neutral IATs used by Snowden and Gray.

Finally, one might ask why the IRAP should be used in research on sexual preferences when other established alternatives exist, such as genital response measures, or are in development, such as eye tracking (Rupp & Wallen, 2007), eye dilation (Rieger & Savin-Williams, 2012), and functional magnetic resonance imaging (Safron et al., 2007). While it remains to be seen whether any of these measures outperform the IRAP or vice versa the IRAP does present a number of intrinsic advantages in that it: (1) requires relatively little training to use, (2) does not require any equipment beyond a basic computer, allowing for large amounts of parallel participant testing, (3) does not have any additional running or equipment costs, (4) is not physically uncomfortable or invasive for participants, (5) has the potential to be developed to be useable with words rather than pictures, and thus is more likely to be acceptable for use in research with minors, and (6) is currently being developed into an online measure for remote data collection, which should allow for large scale data collection in a similar manner to the IAT (Nosek, Greenwald, & Banaji, 2005).

Additionally, and as mentioned previously, the IRAP is in essence a verbal measure (Barnes-Holmes et al., 2010a) which suggests that it (7) may measure a construct that is distinct from, albeit related to, that tapped into by the alternative measures and (8) is more directly comparable with explicit measures. Indeed, these qualities also make the IRAP a good potential candidate to utilize in tandem with genital response or other measures to produce more detailed and perhaps even more accurate results. As such, future research should compare the IRAP with other measures to determine whether they measure the same constructs, their comparative performance and their complementary utility.

In conclusion, we have shown that the IRAP has a powerful ability to identify the sexual orientations of gynephilic and androphilic men, and critically its accuracy in this regard was maintained when measuring separate response biases for male and female stimuli. Indeed, these separate measurements indicated that the response biases of the two sexual orientations targeted here are not strictly orthogonal. This finding raises some interesting questions concerning the variables responsible for the absence of a negative bias among the androphilic men for the female stimuli, especially given that this group rated sex with females negatively on the explicit measure. In any case, these findings provide further support for Snowden et al.’s (2008) conclusion that implicit measurements could prove to be of considerable utility in the study of sexual orientation and sexual preferences.

## Footnotes

1 We use “preference” as a broad term for a number of relativistic responses including those related to sexual orientation. This should not be taken to imply that such responses are volitional.

## References

[CR1] Arkes HR, Tetlock PE (2004). Attributions of implicit prejudice, or “Would Jesse Jackson ‘fail’ the Implicit Association Test?”. Psychological Inquiry.

[CR2] Barnes-Holmes D, Barnes-Holmes Y, Power P, Hayden E, Milne R, Stewart I (2006). Do you really know what you believe? Developing the Implicit Relational Assessment Procedure (IRAP) as a direct measure of implicit beliefs. The Irish Psychologist.

[CR3] Barnes-Holmes D, Barnes-Holmes Y, Stewart I, Boles S (2010). A sketch of the Implicit Relational Assessment Procedure (IRAP) and the Relational Elaboration and Coherence (REC) model. Psychological Record.

[CR4] Barnes-Holmes D, Murphy A, Barnes-Holmes Y, Stewart I (2010). The Implicit Relational Assessment Procedure: Exploring the impact of private versus public contexts and the response latency criterion on pro-white and anti-black stereotyping among white Irish individuals. Psychological Record.

[CR5] Barnes-Holmes D, Murtagh L, Barnes-Holmes Y, Stewart I (2010). Using the Implicit Association Test and the Implicit Relational Assessment Procedure to measure attitudes towards meat and vegetables in vegetarians and meat-eaters. Psychological Record.

[CR6] Barnes-Holmes D, Waldron D, Barnes-Holmes Y, Stewart I (2009). Testing the validity of the Implicit Relational Assessment Procedure (IRAP) and the Implicit Association Test (IAT): Measuring attitudes towards Dublin and country life in Ireland. Psychological Record.

[CR7] Chivers ML, Rieger G, Latty E, Bailey JM (2004). A sex difference in the specificity of sexual arousal. Psychological Science.

[CR8] Chivers ML, Seto MC, Blanchard R (2007). Gender and sexual orientation differences in sexual response to sexual activities versus gender of actors in sexual films. Journal of Personality and Social Psychology.

[CR9] Chivers ML, Seto MC, Lalumière ML, Laan E, Grimbos T (2010). Agreement of self-reported and genital measures of sexual arousal in men and women: A meta-analysis. Archives of Sexual Behavior.

[CR10] Cullen C, Barnes-Holmes D, Barnes-Holmes Y, Stewart I (2009). The Implicit Relational Assessment Procedure (IRAP) and the malleability of ageist attitudes. Psychological Record.

[CR11] Dawson DL, Barnes-Holmes D, Gresswell DM, Hart AJ, Gore NJ (2009). Assessing the implicit beliefs of sexual offenders using the Implicit Relational Assessment Procedure. Sexual Abuse.

[CR12] De Houwer J (2003). The extrinsic affective Simon task. Experimental Psychology.

[CR13] De Houwer J, Wiers R, Stacy A (2006). What are implicit measures and why are we using them?. Handbook of implicit cognition and addiction.

[CR14] De Houwer J, Teige-Mocigemba S, Spruyt A, Moors A (2009). Implicit measures: A normative analysis and review. Psychological Bulletin.

[CR15] Dovidio JF, Kawakami K, Johnson C, Johnson B, Howard A (1997). On the nature of prejudice: Automatic and controlled processes. Journal of Experimental Social Psychology.

[CR16] Dovidio JF, Kawakami K, Smoak N, Gaertner SL, Petty RE, Fazio RH, Brinol P (2009). The roles of implicit and explicit processes in contemporary prejudice. Attitudes: Insights from the new implicit measures.

[CR17] Fawcett T (2006). An introduction to ROC analysis. Pattern Recognition Letters.

[CR18] Fazio RH, Olson MA (2003). Implicit measures in social cognition research: Their meaning and use. Annual Review of Psychology.

[CR19] Fiedler K, Bluemke M (2005). Faking the IAT: Aided and unaided response control on the Implicit Association Tests. Basic and Applied Social Psychology.

[CR20] Freund K, Langevin R, Chamberlayne R, Deosoran A, Zajac Y (1974). The phobic theory of male homosexuality. Archives of General Psychiatry.

[CR21] Freund K, Langevin R, Cibiri S, Zajac Y (1973). Heterosexual aversion in homosexual males. British Journal of Psychiatry.

[CR22] Freund K, Langevin R, Zajac Y (1974). Heterosexual aversion in homosexual males: A second experiment. British Journal of Psychiatry.

[CR23] Friese M, Hofmann W, Wänke M (2008). When impulses take over: Moderated predictive validity of explicit and implicit attitude measures in predicting food choice and consumption behavior. British Journal of Social Psychology.

[CR24] Galdi S, Arcuri L, Gawronski B (2008). Automatic mental associations predict future choices of undecided decision makers. Science.

[CR25] Gawronski B (2009). Ten frequently asked questions about implicit measures and their frequently supposed, but not entirely correct answers. Canadian Psychology.

[CR26] Gawronski B, Bodenhausen GV (2006). Associative and propositional processes in evaluation: An integrative review of implicit and explicit attitude change. Psychological Bulletin.

[CR27] Greenwald AG, Banaji MR (1995). Implicit social cognition: Attitudes, self-esteem, and stereotypes. Psychological Review.

[CR28] Greenwald AG, Nosek BA, Banaji MR (2003). Understanding and using the Implicit Association Test: I. An improved scoring algorithm. Journal of Personality and Social Psychology.

[CR29] Gudjonsson GH, Sigurdsson JF (2000). Differences and similarities between violent offenders and sex offenders. Child Abuse and Neglect.

[CR30] Hughes S, Barnes-Holmes D (2011). On the formation and persistence of implicit attitudes: New evidence from the Implicit Relational Assessment Procedure (IRAP). Psychological Record.

[CR31] Hughes S, Barnes-Holmes D, De Houwer J (2011). The dominance of associative theorizing in implicit attitude research: Propositional and behavioral alternatives. Psychological Record.

[CR32] Israel E, Strassberg DS (2009). Viewing time as an objective measure of sexual interest in heterosexual men and women. Archives of Sexual Behavior.

[CR33] Klein F (1993). The bisexual option.

[CR34] Klein F, Sepekoff B, Wolf TJ (1985). Sexual orientation: A multi-variable dynamic process. Journal of Homosexuality.

[CR35] Lang, P. J., Bradley, M. M., & Cuthbert, B. N. (1997). *International affective picture system (IAPS): Affective ratings of pictures and instruction manual. Technical Report A*-*6.* Gainesville: University of Florida.

[CR36] Layte R, McGee H, Quail A, Rundle K, Cousins G, Donnolly C (2006). The Irish study of sexual health and relationships.

[CR37] Lin CA (1998). Use of sex appeals in prime-time television commercials. Sex Roles.

[CR38] McConnell AR, Leibold JM (2001). Relations among the Implicit Association Test, discriminatory behavior, and explicit measures of racial attitudes. Journal of Experimental Social Psychology.

[CR39] McKenna IM, Barnes-Holmes D, Barnes-Holmes Y, Stewart I (2007). Testing the fake-ability of the Implicit Relational Assessment Procedure (IRAP): The first study. International Journal of Psychology and Psychological Therapy.

[CR40] Nosek BA (2005). Moderators of the relationship between implicit and explicit evaluation. Journal of Experimental Psychology.

[CR41] Nosek BA (2007). Implicit-explicit relations. Current Directions in Psychological Science.

[CR42] Nosek BA, Banaji MR (2001). The Go/No-Go Association task. Social Cognition.

[CR43] Nosek BA, Greenwald AG, Banaji MR (2005). Understanding and using the Implicit Association Test: II. Method variables and construct validity. Personality and Social Psychology Bulletin.

[CR44] O’Ciardha C, Gormley M, Thompson D, Laws DR (2009). Comparing two implicit cognitive measures of sexual interest: A pictorial Stroop task and the Implicit Association Test. Cognitive approaches to the assessment of sexual interest in sexual offenders.

[CR45] O’Ciardha C, Gormley M (2012). Using a pictorial-modified Stroop task to explore the sexual interests of sexual offenders against children. Sexual Abuse.

[CR46] Olson MA, Fazio RH (2001). Implicit attitude formation through classical conditioning. Psychological Science.

[CR47] Olson MA, Fazio RH (2002). Implicit acquisition and manifestation of classically conditioned attitudes. Social Cognition.

[CR48] Power P, Barnes-Holmes D, Barnes-Holmes Y, Stewart I (2009). The Implicit Relational Assessment Procedure (IRAP) as a measure of implicit relative preferences: A first study. Psychological Record.

[CR49] Rieger G, Chivers ML, Bailey JM (2005). Sexual arousal patterns of bisexual men. Psychological Science.

[CR50] Rieger G, Savin-Williams RC (2012). The eyes have it: Sex and sexual orientation differences in pupil dilation patterns. PLoS One.

[CR51] Robertiello G, Terry KJ (2007). Can we profile sex offenders? A review of sex offender typologies. Aggression and Violent Behavior.

[CR52] Roddy S, Stewart I, Barnes-Holmes D (2010). Anti-fat, pro-slim, or both? Using two reaction time based measures to assess implicit attitudes to the slim and overweight. Journal of Health Psychology.

[CR53] Rosenthal AM, Sylva D, Safron A, Bailey JM (2011). Sexual arousal patterns of bisexual men revisited. Biological Psychology.

[CR54] Rullo JE, Strassberg DS, Israel E (2010). Category-specificity in sexual interest in gay men and lesbians. Archives of Sexual Behavior.

[CR55] Rupp HA, Wallen K (2007). Sex differences in viewing sexual stimuli: An eye-tracking study in men and women. Hormones and Behavior.

[CR56] Rydell RJ, McConnell AR (2006). Understanding implicit and explicit attitude change: A systems of reasoning analysis. Journal of Personality and Social Psychology.

[CR57] Safron A, Barch B, Bailey JM, Gitelman DR, Parrish TB, Reber PJ (2007). Neural correlates of sexual arousal in homosexual and heterosexual men. Behavioral Neuroscience.

[CR58] Snowden RJ, Gray NS (2013). Implicit sexual associations in heterosexual and homosexual women and men. Archives of Sexual Behavior.

[CR59] Snowden RJ, Wichter J, Gray NS (2008). Implicit and explicit measurement of sexual preference in gay and heterosexual men: A comparison of priming techniques and the Implicit Association Task. Archives of Sexual Behavior.

[CR60] Timko CA, England EI, Herbert JD, Forman EM (2010). The Implicit Relational Assessment Procedure as a measure of self-esteem. Psychological Record.

[CR61] Vahey NA, Barnes-Holmes D, Barnes-Holmes Y, Stewart I (2009). A first test of the Implicit Relational Assessment Procedure (IRAP) as a measure of self-esteem: Irish prisoner groups and university students. Psychological Record.

[CR62] Weinrich JD, Snyder AJ, Pillard RC, Grant I, Jacobson DL, Robinson SR, McCutchan JA (1993). A factor analysis of the Klein Sexual Orientation Grid in two disparate samples. Archives of Sexual Behavior.

[CR63] Wilson TD, Lindsey S, Schooler TY (2000). A model of dual attitudes. Psychological Review.

